# Gender, ethnic, and socioeconomic differences in access to catheter ablation therapy in patients with atrial fibrillation

**DOI:** 10.3389/fcvm.2022.966383

**Published:** 2023-01-04

**Authors:** Hani Hamade, Ahmad Jabri, Pooja Mishra, Muhammad Umer Butt, Sherin Sallam, Saima Karim

**Affiliations:** ^1^Department of Internal Medicine, The Metrohealth System, Cleveland, OH, United States; ^2^Heart and Vascular Center, The Metrohealth System, Cleveland, OH, United States; ^3^Advocate Heart Institute, Advocate Healthcare, Chicago, IL, United States; ^4^Department of Cardiology, New York University Langone Health, New York, NY, United States; ^5^Department of Internal Medicine, University Hospitals, Cleveland, OH, United States

**Keywords:** atrial fibrillation, gender, healthcare disparity, ablation, ethnicity

## Abstract

**Introduction:**

Female patients, patients from racial minorities, and patient with low socioeconomic status have been noted to have less access to catheter ablation for atrial fibrillation.

**Methods:**

This is a cross-sectional, retrospective study using a large population database (Explorys) to evaluate the gender, racial and socioeconomic differences in access of catheter ablation therapy in patient with atrial fibrillation.

**Results:**

A total of 2.2 million patients were identified as having atrial fibrillation and 62,760 underwent ablation. Females had ablation in 2.1% of cases while males received ablation in 3.4% of cases. Caucasians had ablation in 3.3% of cases, African Americans in 1.5% of cases and other minorities in 1.2% of cases. Individuals on medicaid underwent ablation in 1.6% of cases, individuals on medicare and private insurance had higher rates (2.8 and 2.9%, respectively). Logistic regression showed that female patients (OR 0.608, CI 0.597–0.618, *p* < 0.0001), patients who are African American (OR 0.483, CI 0.465–0.502, *p* < 0.0001), or from other racial minorities (OR 0.343, CI 0.332–0.355, *p* < 0.0001) were less likely to undergo ablation. Patient with medicare (OR 1.444, CI 1.37–1.522, *p* < 0.0001) and private insurance (OR 1.572, CI 1.491–1.658, *p* < 0.0001) were more likely to undergo ablation.

**Conclusion:**

Female gender, racial minorities, low socioeconomic status are all associated with lower rates of catheter ablation in management of atrial fibrillation.

## Introduction

Atrial fibrillation (AF) is one of the most common arrhythmias worldwide, and the rates of atrial fibrillation incidence and prevalence are expected to rise further in upcoming years (1). Atrial fibrillation is usually managed either medically or *via* intervention such as cardioversion and catheter ablation (CA), and there is increasing evidence to suggest that disparities exists in AF management between people from different gender, ethnicities, and socioeconomic status (2, 3). Multiple studies have suggested that women tend to receive CA less despite having a worse symptom burden (2). In a review of administrative encounter data for medicare beneficiaries between 2010 and 2011 (*N* = 517,941), Bhave et al. showed that female patients were less likely to undergo CA with an adjusted hazard ratio of 0.70 with a 95% Confidence Interval (CI) of 0.63–0.79 (4). The largest study to date conducted by Patel et al. reviewed a large population database (Nationwide Inpatient Sample) which included 3,508,122 patients between 2000 and 2012 and found that female patients were also less likely to undergo CA compared to men (5). Such findings are not limited to the United States and have also been reported in Spain, China, Japan, and other countries (6–9). Similarly, many studies also suggest that African Americans, Hispanics, and other racial and ethnic minorities have lower rates of undergoing CA (4, 10–13). In a cross-sectional analysis conducted on a large sample of patients in Florida (*N* = 923,590), Tamariz et al. showed that African American and Hispanic patients were less likely to undergo CA (13). Eberly et al. analyzed a sample (*N* = 109,221) from the Optum Clinformatics Data Mart and found that African American patients were less likely to receive CA and patients of Latin origin were less likely to receive CA when rhythm control is pursued (10). Such differences in access to CA should be evaluated and addressed in order to be able to ensure equality in atrial fibrillation management. The aim of this study is to provide an updated evaluation of gender, racial and socioeconomic differences in use of catheter ablation in the treatment of atrial fibrillation in one of the largest population databases in the United States.

## Methods

This study is a cross-sectional study using Explorys (IBM Watson Health; Cleveland, OH, USA), de-identified HIPAA compliant, large population-based database *via* electronic health record (EHR). Explorys obtains de-identified data from participating healthcare systems caring for an aggregate large patient population with countrywide distribution, and collects data from various sources such as EHR, billing systems, laboratory test systems etc. The information collected from Explorys in compliant with Health Insurance Portability and Accountability Act (HIPAA) and Health Information Technology for Economic and Clinical Health Act (HITECH). Data was obtained from the de-identified and pooled electronic health records of over 63 million patients as of January 2022 using the Systematized Nomenclature for Medicine—Clinical Terms (SNOMED-CT) for medical diagnoses and procedures. Patients with atrial fibrillation were identified with the search term, “atrial fibrillation” and ablation was found using the search term “ablation procedure for arrhythmia”. Ethnicity was obtained and divided into three categories which included Caucasian, African American, and Other (other racial minorities). Insurance provider (medicare, medicaid, private insurances) were used as a surrogate for socioeconomic status (14). For each gender, ethnicity and insurance type, the number of ablations were obtained, and the rates of ablations were calculated accordingly. Finally, a binary logistic regression was performed to evaluate the adjusted effect of these variables on likelihood of undergoing ablation.

## Results

A total of 2.2 million patients were identified as having atrial fibrillation, with 46.3% being female. Up to 76.8% were Caucasian with 7.7% being African American and 15.5% being from other racial minorities. Medicare was the most common insurance type (54.9%), followed by private insurance (41.4%) and then medicaid (3.6%). Table 1 displays the demographic distribution of atrial fibrillation patients within Explorys. A total of 62,760 patients underwent ablation with 34.4% being female and 65.6% being male. When comparing rates of ablation among genders, females were noted to have had ablation in 2.1% of cases while males received ablation in 3.4% of cases. Caucasians had the highest rate of ablation (3.3%) while African Americans (1.5%) and other minorities (1.2%) had lower rates. Individuals on medicaid underwent ablation in 1.6% of cases while individuals on medicare and private insurance had higher rates (2.8 and 2.9%, respectively). Table 2 displays the demographic distribution of patients who received ablation and those who did not receive ablation.

**Table 1:** Demographic distributions of all patients with atrial fibrillation.

**Variables**		**Atrial fibrillation**	**Percentage (%)**
Gender	Male	1,201,370	53.7
	Female	1,035,210	46.3
Race	Caucasian	1,718,310	76.8
	African American	171,430	7.7
	Other	346,840	15.5
Insurance	Medicaid	80,900	3.6
	Medicare	1,228,930	54.9
	Private	926,750	41.4
Total		2,236,580	100.0

**Table 2:** Demographic distribution of all patients with atrial fibrillation who have undergone catheter ablation.

**Variables**		**Ablation**	**Percentage (%)**	**No ablation**	**Percentage (%)**	**Total**
Gender	Male	41,160	3.4	1,160,210	96.6	1,201,370
	Female	21,600	2.1	1,013,610	97.9	1,035,210
Race	Caucasian	56,100	3.3	1,662,210	96.7	1,718,310
	African American	2,540	1.5	168,890	98.5	171,430
	Other	4,120	1.2	342,720	98.8	346,840
Insurance	Medicaid	1,260	1.6	79,640	98.4	80,900
	Medicare	34,550	2.8	1,194,380	97.2	1,228,930
	Private	26,950	2.9	899,800	97.1	926,750
Total		62,760	2.8	2,173,820	97.2	2,236,580

Table 3 demonstrate the result of the logistic regression model used to evaluate the effect of each of these demographic variables on likelihood of receiving ablation. Unsurprisingly, the model only explained 2% (Nagelkerke R2) of the variance in ablation use which is expected given the fact that several variables were not available to be included. Women were less likely than men to undergo ablation with an odds ratio (OR) of 0.608 (CI 0.597–0.618, *p*-value < 0.0001). African American and patients from other racial minorities were also less likely to undergo ablation compared to their Caucasian counterparts (OR 0.483 CI 0.465–0.502, *p* < 0.0001 and OR 0.343 CI 0.332–0.355, *p* < 0.0001, respectively). Patients on medicare and private insurance were also more likely to undergo ablation compared to patients on medicaid (OR 1.444 CI 1.37–1.522, *p*-value < 0.0001 and OR 1.572 CI 1.491–1.658, *p*-value < 0.0001).

**Table 3:** Logistic regression evaluating the effects of the different demographic variables on likelihood of undergoing catheter ablation.

**Variables**	**Exp(B)**	**95% CI for Exp(B)**	***p*-value**
Medicaid	–	–	< 0.0001
Medicare	1.444	1.37–1.522	< 0.0001
Private	1.572	1.491–1.658	< 0.0001
Male	–	–	< 0.0001
Female	0.608	0.597–0.618	< 0.0001
Caucasian	–	–	< 0.0001
African American	0.483	0.465–0.502	< 0.0001
Other	0.343	0.332–0.355	< 0.0001
Constant	0.028	–	< 0.0001

Exp(B), adjusted odds ratio; CI, confidence interval.

## Discussion

While cardiovascular disease remains the most common cause of morbidity and mortality for women, there have been significant sex differences in mortality and management favoring male patients (15). When it comes to atrial fibrillation management, there is an increasingly growing body of literature suggesting that women are receiving less CA therapy (2, 16). Such findings are redemonstrated in our study which indicates that the differences in the rates of CA use between male and female patients remain observable. This pattern is particularly interesting given that women are equally likely if not more likely to have atrial fibrillation in comparison to men. Women also have been shown to have worse symptom burden and quality of life compromise compared to men (16–18). Additionally, they have worse clinical outcomes with antiarrhythmic medications, promoting CA as an especially viable therapeutic option (18). The reasons behind the lower rates of CA use in women remain incompletely understood but multiple potentially contributing factors have been discussed. Scheuermeyer et al. suggest that women are more likely to present with atypical symptoms in comparison to men, which may lead to lower detection rates or more conservative treatment (19). Additionally, women are usually referred later and less frequently for ablation in comparison to men, and have more comorbidities at time of AF management is initiated (18, 20, 21). There is also evidence to suggest that women have a higher rate of atrial fibrillation recurrence and higher likelihood of complications from CA, although these findings have been inconsistent. In a sample of 21,091 patients who underwent CA between 2007 and 2011, Kaiser et al. noted that women were more likely to undergo an AF-related hospital admission and to have post-procedural complications after undergoing CA (22). Similarly, Ngo et al. analyzed a sample of 35,211 patients who underwent CA between 2008 and 2017 and found that women had a 25% higher risk of post-procedural complications (23). In contrast, Russo et al. reported results from the CABANA trial in 2021 and did not find significant differences between male and female participants in terms of major complications (24). Of note, most reviews and trials have noted that older age and more comorbidities at time of ablation may also be playing a significant role in these findings (18, 25, 26). Personal choice may also play a role, with Takigawa et al. reporting that women were more likely to forego CA, particularly in the case of repeat ablation (27). Understanding women's needs, presentations, risk factors, treatment responses and complication rates may help shed light on the barriers that they face in terms of access to CA as differences in these factors can affect the decision to undergo CA, whether from the side of the patient or provider. Increasing effort is being made in this regard, and continues to be needed to understand and bridge this gap in care between genders.

Our study also shows a significant difference between Caucasian and non-Caucasian patients in their rates of atrial fibrillation ablation. This is consistent with other studies which have shown that African American, Hispanic and Asian American were all less likely to undergo CA in comparison to their Caucasian counterparts (4, 10, 11, 28). Many racial and ethnic minorities have numerous reasons why they would benefit from atrial fibrillation ablation. African American and other racial minority patients with atrial fibrillation have been shown to have a worse quality of life and a higher stroke risk and mortality (3, 29, 30). Additionally, the CABANA trial has shown that racial minorities overall have worse outcomes with antiarrhythmic drug in comparison with CA, which may suggest that CA can be a viable treatment modality (12, 31, 32). Such patterns are not limited to CA, with studies suggesting that racial and ethnic minorities also have lower access to other atrial fibrillation treatment modalities (33, 34). Essien et al. showed that African Americans were less likely to receive anticoagulation when indicated. This is still noted when adjusting for socioeconomic status. Additionally, Golwala et al. showed that African American and Hispanic patients were more likely to be treated with rate control compared to rhythm control, although the lower tolerance for anti-arrhythmic drugs may partly explain this finding (30). Symptom burden plays a role in the decision to pursue CA, however limited evidence seems to point to the fact that non-Caucasian patients are no less symptomatic than their Caucasian counterparts (30, 35). Such differences between Caucasian and non-Caucasian patients need to be better understood in order to improve access to CA and other atrial fibrillation management modalities.

Lastly, medicaid patients were less likely to receive CA. Insurance types were used as a surrogate for socioeconomic status in this study and the aforementioned finding is consistent with the literature indicating that patients with lower socioeconomic status were less likely to receive CA (10). The reasons behind this may go beyond the financial aspect. In a study conducted in Norway, Olsen et al. showed a significant positive association between socioeconomic status and rates of CA. They argue that health literacy and patient preferences may be important factors in the decision to undergo ablation and may be preventing patients with lower socioeconomic status from undergoing ablation (36). Eberly et al. showed similar findings. The authors note that clinician bias may be an important factor with physicians avoid offering ablation to patients with lower socioeconomic status due to concern that they may be unable to understand and navigate the procedure and its implications (10).

The main strength of this study is the large patient sample of 2.2 million patients with atrial fibrillation. To our knowledge, this is the largest patient sample used to study gender, ethnic, and socio-economic differences since the study conducted by Patel et al. in 2013. The study highlights that such differences are still observable across multiple systems and that further work is needed to bridge the gaps. The study also has multiple limitations, which are mostly related to the limited nature of the data extracted from a de-identified, large population database. Many factors that may affect CA were difficult to assess accurately using the nature of the database such as symptom burden, quality of life, average age at onset, and other clinical factors. Other details such as additional insurances that may not be listed in the database could also not be included. Additionally, the electronic health record codes used to document diagnoses and treatment modalities may not always show the true diagnosis and are subject to human charting error. Another challenge is that the inclusion of comorbidities and prior treatment attempts in the analysis was limited as establishing accurate temporal relations with the catheter ablation therapy and measuring their impact on decision making was challenging given the cross-sectional approach of the study. Understanding the management history and treatment attempts made prior to ablation consideration is important and factors into management decisions, however in light of the limitation noted this was not accounted for in this analysis. The cross-sectional approach also makes it difficult to evaluate the trend of use of CA across the years among the different groups. This could be addressed through cohort studies that follow patients from the initial diagnosis atrial fibrillation. Lastly, the study includes only patients included in the database which itself includes a specific number of healthcare centers, and therefore may not be entirely generalizable to the national population. However, the large number of patients in the database may offset some of these limitations.

## Conclusion

Among patients with atrial fibrillation, there are significant gender, racial, and socioeconomic differences in utility of catheter ablation. Women, racial and ethnic minorities, and individuals with lower socioeconomic status were all less likely to receive ablation. There is a persistent need to understand the various causes that result in gender, racial, ethnic, and socioeconomic disparities to ensure a safe and equitable access for care among all patients suffering from atrial fibrillation.

## Data availability statement

The raw data supporting the conclusions of this article will be made available by the authors, without undue reservation.

## Author contributions

SK, HH, PM, and AJ conceived the project and study design. HH and PM performed the data collection and data cleaning. HH and MB performed the data analysis. AJ and HH wrote the first draft of the manuscript. SK, PM, and MB wrote sections of the manuscript. All authors contributed to revision, editing, and proof-reading of the manuscript. All authors contributed to the article and approved the submitted version.
